# Predictive Factors of Long-Term Stay in the ICU after Cardiac Surgery: Logistic CASUS Score, Serum Bilirubin Dosage and Extracorporeal Circulation Time

**DOI:** 10.21470/1678-9741-2016-0072

**Published:** 2017

**Authors:** Marcio Fernandes Pimentel, Marcelo José Ferreira Soares, Jamil Alli Murad Junior, Marcos Aurelio Barboza de Oliveira, Fernanda Luiza Faria, Vinicius Zani Faveri, Yuzo Iano, Rodrigo Capobianco Guido

**Affiliations:** 1 Faculdade de Medicina de São José do Rio Preto (FAMERP), São José do Rio Preto, SP, Brazil.; 2 Santa Casa Votuporanga, Votuporanga, SP, Brazil.; 3 Faculdade de Engenharia Elétrica e de Computação da Universidade Estadual de Campinas (FEEC-Unicamp), Campinas, SP, Brazil.; 4 Instituto de Biociências, Letras e Ciências Exatas da Universidade Estadual Paulista (IBILCE-UNESP), São José do Rio Preto, SP, Brazil.

**Keywords:** Intensive Care Unit, Cardiovascular Surgical Procedures, Organ Dysfunction Scores, Bilirubin, Extracorporeal Circulation

## Abstract

**Objective:**

To test the capacity of the Logistic CASUS Score on the second postoperative day, the total serum bilirubin dosage on the second postoperative day and the extracorporeal circulation time, as possible predictive factors of long-term stay in Intensive Care Unit after cardiac surgery.

**Methods:**

Eight-two patients submitted to cardiac surgery with extracorporeal circulation were selected. The Logistic CASUS Score on the second postoperative day was calculated and bilirubin dosage on the second postoperative day was measured. The extracorporeal circulation time was also registered. Patients were divided into two groups: Group A, those who were discharged up to the second day of postoperative care; Group B, those who were discharged after the second day of postoperative care.

**Results:**

In this study, 40 cases were listed in Group A and 42 cases in Group B. The mean extracorporeal circulation time was 83.9±29.4 min in Group A and 95.8±29.31 min in Group B. Extracorporeal circulation time was not significant in this study (*P*=0.0735). The level of *P* significance of bilirubin dosage on the second postoperative day was 0.0003 and an area under the ROC curve of 0.708 with a cut-off point at 0.51 mg/dl was registered. The level of *P* significance of Logistic CASUS Score on the second postoperative day was 0.0001 and an area under the ROC curve of 0.723 with a cut-off point at 0.40% was registered.

**Conclusion:**

The Logistic CASUS Score on the second postoperative day has shown to be better than the bilirubin dosage on the second postoperative day as a predictive tool for calculating the length of stay in intensive care unit during the postoperative care period of patients. Notwithstanding, extracorporeal circulation time has failed to prove itself as an efficient tool to predict an extended length of stay in intensive care unit.

**Table:** 

Abbreviations, acronyms & symbols	
Apache II	= Acute Physiology And Chronic Health Evaluation Score II
BLR2PO	= Bilirubin dosage on the second postoperative day
CABG	= Coronary artery bypass grafting surgery
CASUS	= Cardiac Surgery Score
ECT	= Extracorporeal circulation time
ICU	= Intensive care unit
Log-CASUS	= Logistic Cardiac Surgery Score
LOG2PO	= Logistic CASUS Score on the second postoperative day
PO	= Postoperative period
ROC	= Receiver operating characteristic
SAPS	= Simplified Acute Physiology Score
SOFA	= Sequential Organ Failure Assessment Score

## INTRODUCTION

The science of treating ailing patients goes far beyond the medical capacity to impose specific therapeutic measures for each illness, reaching the necessity to know how to anticipate severity, mortality and possible aggravations any disease might carry. Knowing how to make such predictions capacitates us in a way that our intervention becomes more individualized and more efficient for each patient. This is why prognostic scores are widely used tools in the day-to-day practice of many medical specialties, as much in the scope of preoperative care as postoperative care in the intensive care units.

The postoperative period (PO) of cardiac surgery is characterized by a great instability of the hemodynamic conditions of the patients, mainly of those submitted to extracorporeal circulation. This procedure contributes for the highest risk of aggravations and complications in several organ systems, deriving from a systemic inflammatory reaction^[1]^, which consequently may lead to a longer stay in the intensive care unit (ICU), as well as the highest mortality able to be predicted in mortality scores in the ICU, such as Acute Physiology And Chronic Health Evaluation Score II (Apache II), Simplified Acute Physiology Score (SAPS) and Sequential Organ Failure Assessment Score (SOFA). Simultaneously, these scores do not contemplate the conditions of the cardiac patients submitted to surgeries with extracorporeal circulation, aside from being less accurate in predicting postoperative mortality, when compared to Logistic Cardiac Surgery Score (CASUS) -Log-CASUS^[2]^.

The Log-CASUS is a daily mortality score specifically related to patients in extracorporeal circulation during cardiac surgery postoperative care; it is a method of easy stratification and implementation at a patient's bedside^[3,4]^, which can be done on the internet^[5,6]^.

The objective of this cohort retrospective study is to investigate the correlation between the Log-CASUS score on the second postoperative day (LOG2PO) and the total serum bilirubin dosage in the same period (BLR2PO), associated to the extracorporeal circulation time (ECT) as possible predictive factors of extended stay in the ICU.

## METHODS

This study has been submitted to and approved by the Ethics and Research Committee of FAMERP, São José do Rio Preto Medical School, State of São Paulo, Brazil, under number 054254/2016, on June 9^th^, 2016. The patients were randomly selected, among those were operated in last 6 months in this service. Eight-two patients, whose full clinical and laboratorial data was available during the research period, were included in the study, clinical and laboratorial data regarding the postoperative care of adult patients submitted to cardiac surgeries with extracorporeal circulation was collected, carried out in the teaching hospital of São José do Rio Preto Medical School - Hospital de Base da Fundação Faculdade de Medicina de São José do Rio Preto-FURNFARME. Patients were divided into two groups: Group A - those who were discharged up to the second day of postoperative care; Group B - those who were discharged after the second day of postoperative care. The assessed surgeries were myocardial revascularization with extracorporeal circulation, valve replacement and plastic surgery, as well as aorta and congenital surgery. The surgeries which consider two or more types, among those above mentioned, were classified as combined surgeries.

**Fig. 1:**
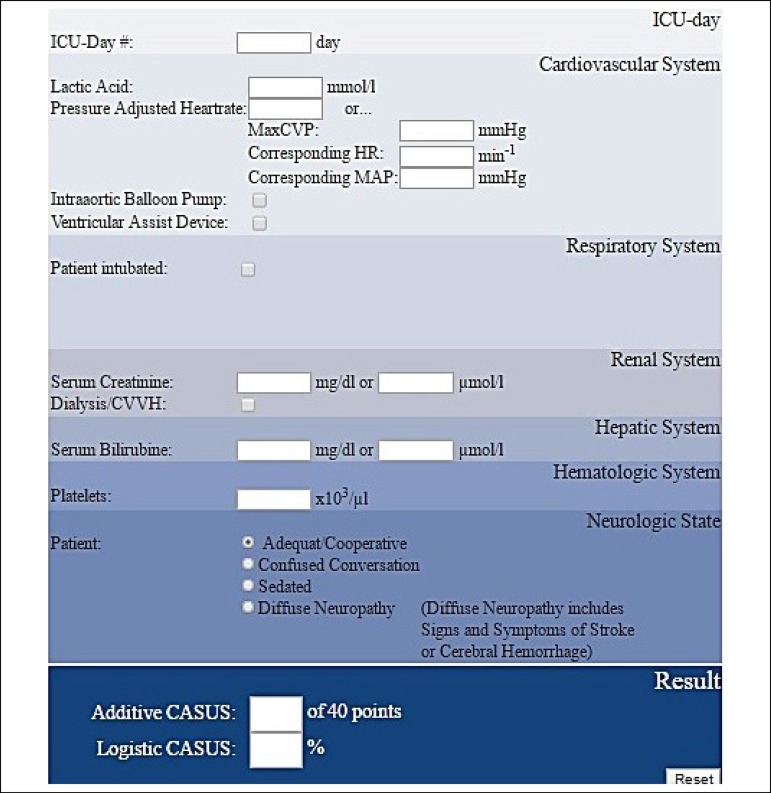
CASUS Online Calculations (http://www.cardiac-icu.org/Online-Calculation.html) ^[5]^.

All surgeries were performed with the Braile^®^ extracorporeal circulation, under mild hypothermia with perfusion of the ascending aorta and bicaval venous drainage, excepting in coronary artery bypass grafting surgery (CABG) - where an atrial venous drainage with two-stage cannula was performed. The combined antegrade and retrograde low-volume continuous isothermic blood cardioplegia with a Braile Biomedica^®[7]^ preservation solution was the chosen myocardial protection technique for these surgeries. The BLR2PO was used, and the LOG2PO for the same period was calculated. This was performed online through www.cardiac-icu.org/Online-Calculation.htm^[5]^, as shown in Figure 1. The ECT for each surgery was also registered.

### Statistical Analysis

Regarding the baseline characteristics of the groups, the Fisher exact test (chi-square) was performed.

Data was presented in mean ± standard deviation, median (percentil 25-75) or absolute value (%), adequately, with the Kolmogorov-Smirnoff test for normality assessment of variables used.

The statistical softwares used were both MedCalc V14 and StatDirect. Group A variables BLR2PO, LOG2PO and ECT were compared to Group B respective variables, then the accuracy of such parameters was analyzed by Receiver Operator Characteristic (ROC) Curves, in order to determine the cut-off point for those variables.

Value *P*<0.05 was defined as significant.

## RESULTS

The average age in Group A was 57.9±10.7 and 60.1±10.8 in Group B. The gender distribution in Group A was 60% male and 40% female, whereas in Group B the distribution was 54.7% and 45.3%, respectively (Table 1).

**Table 1:** Characteristics of Group A and B.

	Group A 40 Patients	Group B 42 Patients
Average age (years)	57.95±10.7	60.14±10.81
Female	16 (40%)	19 (45.3%)
Male	24 (60%)	23 (54.7%)

Regarding the surgeries performed, a tendency for the groups to be heterogeneous regarding aortic surgery was noted, without statistical significance (*P*=0.054) (Table 2).

**Table 2:** Assessed surgeries (Group A vs. Group B).

Surgeries	Group A 40 Patients	Group B 42 Patients	*P* value
CABG	27 (67.50%)	23 (54.70%)	0.369
Valve	11 (27.50%)	12 (28.50%)	0.362
Combined	1 (2.50%)	5 (11.90%)	0.202
Congenital	1 (2.50)	__	0.488
Aorta	__	2 (4.90%)	0.054

CABG=coronary artery bypass grafting surgery

The mean ECT was 83.9± 29.4 minutes in Group A, and 95.8±29.31 minutes in Group B.

The mean values of bilirubin (percentile) on the second day of PO (BLR2PO) were 0.34 (0.28-0.45) mg/dl in Group A, and 0.52 (0.37-0.94) mg/dl in Group B. The mean value of the Log-CASUS (percentile) on the second day of PO (LOG2PO) was 0.26 (0.21-0.37) for Group A, and 0.50 (0.29-1.60) for Group B (Table 3).

**Table 3:** Values of variables in Group A and B.

	Group A 40 Patients	Group B 42 Patients
Mean ECT (min)	83.9±29.4	95.8±29.31
BLR2PO (mg/dl)	0.34 (0.28-0.45)	0.52 (0.37-0.94)
LOG2PO (%)	0.26 (0.21-0.37)	0.50 (0.29-1.60)

The variables ECT, BLR2PO and LOG2Po were tested in an isolated manner as predictive factors of the length of stay in the ICU to be more than 48h following cardiac surgery.

**Fig. 2:**
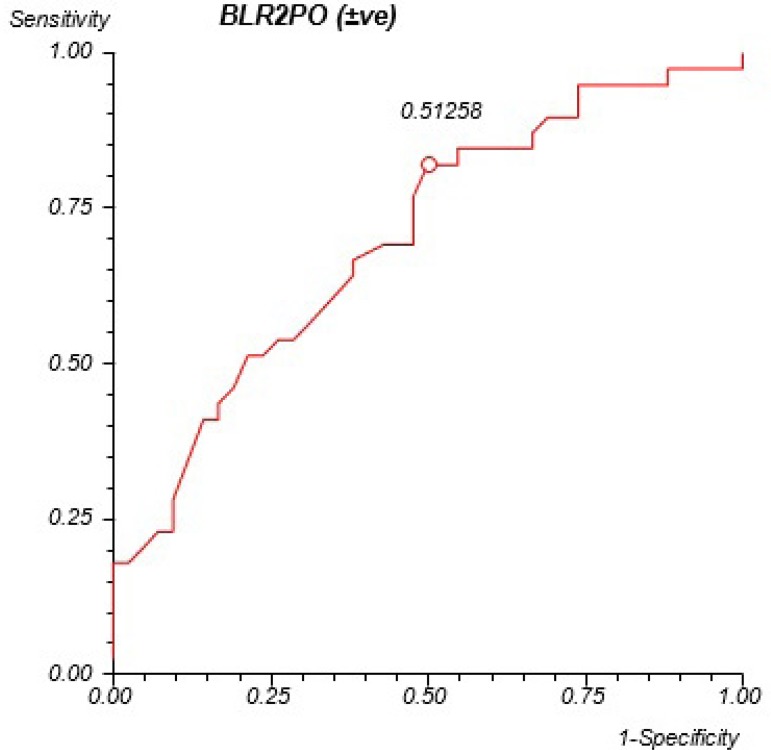
ROC curve for BLR2PO. Area under the ROC curve=0.708; P=0.003; Sensitivity=82.05%; Specificity=51.16%.

**Fig. 3:**
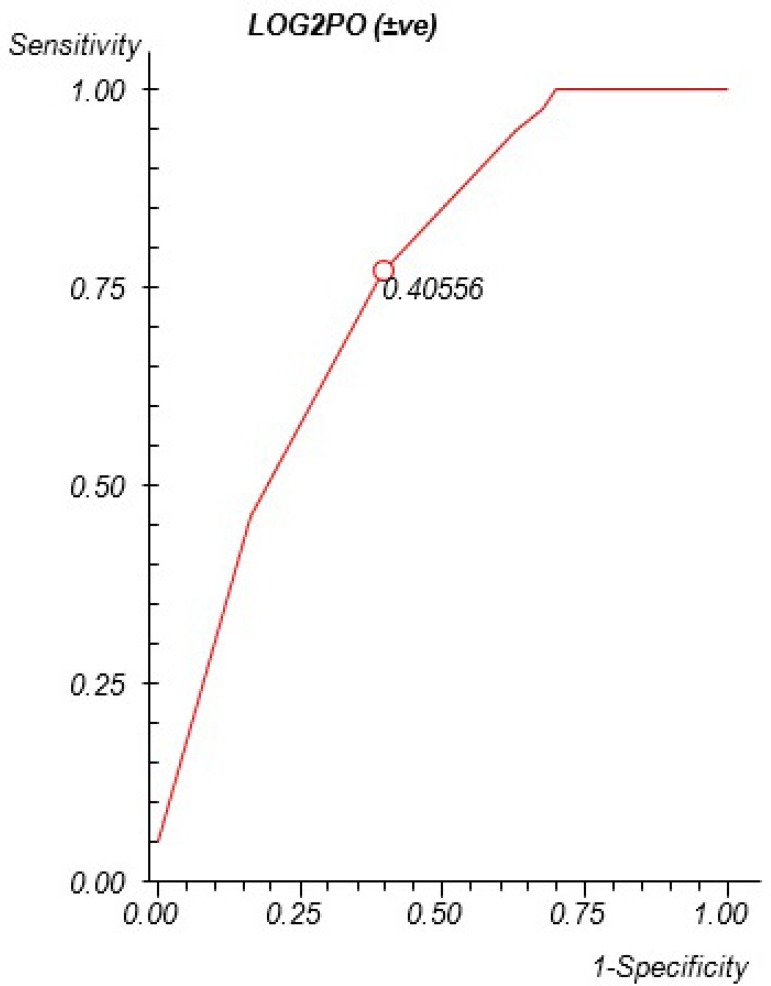
ROC curve for LOG2PO. Area under the ROC curve=0.723; P=0.0001; Sensitivity=74.36%; Specificity=62.79%.

A level of *P* significance of 0.0735 for ECT, and an area under the ROC curve of 0.612 were observed. For BLR2PO, a level of *P* significance of 0.0003 was registered, as well as an area under the ROC curve of 0.723, with the cut-off point at 0.51 mg/dl (Figure 2). Concerning LOG2PO, 0.0001 was the level of *P* significance found, 0.723 of area under the ROC curve, and the cut-off point at 0.40% (Figure 3).

## DISCUSSION

Being able to anticipate results in cardiac surgical procedures is a great challenge. To find means that can equate all the complexities related to cardiac surgeries and sum the levels of severity of illness for each patient is a feat far too distant from our current capacity. Meanwhile, we try to adjust those answers by means of specific tools that aid the universe of cardiovascular surgery. For this, LOG2PO, BLR2PO and ECT were tested as possible predictive factors of length of stay in the ICU during postoperative care.

In this article, correlations among three distinguishable variables were conducted, with the goal to assess their relevance either in early discharge from the ICU (up to the second day of postoperative care) or in late discharge (after the second day of postoperative care). Clear differences amongst the samples of variables collected from each group were registered, with the mean values of all the variables studied higher in Group B, namely ECT, BLR2PO and LOG2PO. The higher mean occurred in the group of patients with longer length of stay in the ICU.

The Log-CASUS assesses six organ systems accrued with the variable "day of admission in the ICU" and, contrary to the remaining scores, it punctuates the presence of circulatory assistance devices and the use of an intra-aortic balloon^[3]^. As a mortality predictive factor, the Log-CASUS has proved to be quite a straightforward tool, reliable and superior to SOFA^[2]^. Hence, the possibility of the LOG2PO to predict beyond the risk of daily mortality was tested, along the lines of extended length of stay in postoperative care.

The measure of total serum bilirubin on the second day of postoperative care (BLR2PO) was tested as variable of longer length of stay in the ICU, from observation, within clinical practice, of a considerable increase in the length of hospitalization both in the worst cases and those with longer ECT; such occurrence was also established by other authors^[8]^. To be also considered is the fact that the isolated total serum bilirubin dosage in the second day of postoperative care is an identifying factor of patients with greater risk of mortality within this period^[9]^. The results indicated that BLR2PO could predict a longer permanence in ICU. However, the calculation of the LOG2PO, which is obtained through other variables, among them the BLR2PO itself, was more expressive in predicting the long stay in the ICU as well as two days, than the simple measure of serum bilirubin.

The ECT was clearly higher in Group B. Indubitably, we understand this was due to the fact that the number of combined surgeries and Group B's aorta surgeries (16.8% of cases) were greater than those in Group A (2.5% of cases); these are known complex procedures which normally demand more extracorporeal circulation time. Meanwhile, the ECT did not have sufficient discriminative power in predicting an extended length of stay.

On the other hand, the definition of extended length of stay for patients in cardiac procedure postoperative care is quite controversial in the literature. Silberman et al.^[10]^ determined a prolonged hospitalization that which registers a period longer than 48h, and noted that 73% of their patients were in this group. Lagercrantz et al.^[11]^ construed an extended length of stay in the ICU to be superior to 10 days, and observed that only 3.5% of their patients were in this group. When following protocol in the routine of our institution, we consider an ideal postoperative recovery, not aggravated, to be one which occurs within the 48h period after surgery. Thus, our group of extended length of stay (Group B), following Silberman et al.^[10]^ criteria, comprised 51% of the cases studied.

### Limitations of the Study

This study was carried out with a small number of patients, and the severity of the included cases was not quantified, nor organized per group. The only factor quantified was the length of stay in the intensive care unit for both groups.

## CONCLUSION

The distinguishable analysis of the influence of each variable in anticipating the discharge from the ICU up to or after the second day of PO has shown the most relevant variable to be LOG2PO, as the area under the respective ROC curve found from the Log-CASUS parameter was 0.723, *i.e.*, the greatest among those analyzed. Hence, proving the viability of using such variable to estimate whether the postoperative length of stay in the ICU of these patients will be a tough task. On the other hand, the ECT has not proved to be a good predictive factor for longterm stay in the ICU.

For future works, there is an intention to, beyond the enlargement of the database, establish the correlation of the three parameters studied, by means of distinct numericalstatistical techniques, in order to obtain more relevant and comprehensive results for the referred prediction.

**Table:** 

Authors' roles & responsibilities	
MFP	Conception and design study; realization of operations and/or trials; analysis and/or data interpretation; manuscript redaction or critical review of its content; final manuscript approval
MJFS	Realization of operations and/or trials; critical review of the manuscript; final manuscript approval
JAMJ	Analysis and/or data interpretation; final manuscript approval
MABO	Analysis and/or data interpretation; statistical analysis; final manuscript approval
FLF	Data collection; final manuscript approval
VZF	Analysis and/or data interpretation; final manuscript approval
YI	Critical review of its content; final manuscript approval
RCG	Analysis and/or data interpretation; statistical analysis; final manuscript approval

## References

[1] Levy JH, Tanaka KA (2003). Inflammatory response to cardiopulmonary bypass. Ann Thorac Surg.

[2] Doerr F, Badreldin AM, Heldwein MB, Bossert T, Richter M, Lehmann T (2011). A comparative study of four intensive care outcome prediction
models in cardiac surgery patients. J Cardiothorac Surg.

[3] Doerr F, Heldwein MB, Bayer O, Sabashnikov A, Weymann A, Dohmen PM (2015). Inclusion of 'ICU-day' in a logistic scoring system improves
mortality prediction in cardiac surgery. Med Sci Monit Basic Res.

[4] Badreldin AM, Kroener A, Heldwein MB, Doerr F, Vogt H, Ismail MM (2010). Prognostic value of daily cardiac surgery score (CASUS) and its
derivatives in cardiac surgery patients. Thorac Cardiovasc Surg.

[5] Hekmat K Online calculation of the cardiac intensive care score.

[6] Exarchopoulos T, Charitidou E, Dedeilias P, Charitos C, Routsi C (2015). 2015 Scoring systems for outcome prediction in a cardiac surgical
intensive care unit: a comparative study. Am J Crit Care.

[7] Braile DM (1992). Como eu faço: cardioplegia sangüínea
isotérmica retrógrada de baixo volume. Rev Bras Cir Cardiovasc.

[8] Sabzi F, Faraji R (2015). Liver function tests following open cardiac
surgery. J Cardiovasc Thorac Res.

[9] Collins JD, Bassendine MF, Ferner R, Blesovsky A, Murray A, Pearson DT (1983). Incidence and prognostic importance of jaundice after
cardiopulmonary bypass surgery. Lancet.

[10] Silberman S, Bitran D, Fink D, Tauber R, Merin O (2013). Very prolonged stay in the intensive care unit after cardiac
operations: early results and late survival. Ann Thorac Surg.

[11] Lagercrantz E, Lindblom D, Sartipy U (2010). Survival and quality of life in cardiac surgery patients with
prolonged intensive care. Ann Thorac Surg.

